# Emerging protein degradation strategies: expanding the scope to extracellular and membrane proteins

**DOI:** 10.7150/thno.62686

**Published:** 2021-07-13

**Authors:** Jiayi Lin, Jinmei Jin, Yiwen Shen, Lijun Zhang, Gang Gong, Huiting Bian, Hongzhuan Chen, Dale G. Nagle, Ye Wu, Weidong Zhang, Xin Luan

**Affiliations:** 1 Institute of Interdisciplinary Integrative Medicine Research and Shuguang Hospital, Shanghai University of Traditional Chinese Medicine, Shanghai, 201203, China; 2 School of Pharmacy, Second Military Medical University, Shanghai, 201203, China; 3 Department of Biomolecular Sciences and Research of Institute of Pharmaceutical Sciences, School of Pharmacy, University of Mississippi, University, MS, 38677-1848, USA

**Keywords:** targeted protein degradation (TPD), extracellular and membrane proteins, LYTAC, AbTAC

## Abstract

Classic small molecule inhibitors that directly target pathogenic proteins typically rely on the accessible binding sites to achieve prolonged occupancy and influence protein functions. The emerging targeted protein degradation (TPD) strategies exemplified by PROteolysis TArgeting Chimeras (PROTACs) are revolutionizing conventional drug discovery modality to target proteins of interest (POIs) that were categorized as “undruggable” before, however, these strategies are limited within intracellular POIs. The novel new degrader technologies such as LYsosome-TArgeting Chimaeras (LYTACs) and Antibody-based PROTACs (AbTACs) have been successfully developed to expand the scope of TPD to extracellular and membrane proteins, fulfilling huge unmet medical needs. Here, we systematically review the currently viable protein degradation strategies, emphasize that LYTACs and AbTACs turn a new avenue for the development of TPD, and highlight the potential challenges and directions in this vibrant field.

## Introduction

Remarkable advances in human genetic studies have revealed a broad spectrum of protein targets that associate with disease progression 1, 2. The classical small molecule inhibitor paradigm has been pursued worldwide through large-scale screening and structure-based optimization by academic and industry groups. Nevertheless, traditional drug discovery approaches usually require accessible binding sites and measurable biochemical index of target proteins, thus, only a small portion of human proteome is pharmaceutically druggable 3. The majority of disease-causing proteins, including scaffolding proteins, transcription factors, and other non-enzymatic proteins are rendered as undruggable targets for a long time 4, 5.

Targeted protein degradation (TPD) has emerged to be a powerful tool to handle these undruggable targets and exhibits significant therapeutic benefit over standard small-molecule inhibition strategy 6. A full degradation of disease-causing proteins provides the chance for ablation of target protein as well as all of its associated biological functions. It has been demonstrated that small molecules can induce protein degradation at the post-translational level, which occur with estrogen receptor alpha (ERα), inhibitor of apoptosis protein (IAP), androgen receptor (AR) and others 7-9. However, this strategy has limited applications because there is rare ligand possessing degradation potency and it is wrought with great uncertainties. Recently, TPD strategies have expanded to include new modalities: PROteolysis Targeting Chimeras (PROTACs) 10, autophagy-targeting chimeras (AUTACs) 11, autophagosome-tethering compounds (ATTECs) 12, molecular glue degraders 13, 14, the degradation tag (dTAG) system 15, 16, and Trim-Away 17, 18, etc. Among them, PROTAC is the most advanced degradation strategy with two oral PROTACs (ARV-110 and ARV-471) exhibiting encouraging outcomes in Phase Ⅰ clinical trials 19. However, these novel degrader technologies have only been applied to intracellular protein targets, the extracellular and membrane proteins that consist of more than 40% of human proteome still lack of effective therapeutic degraders 20.

To broaden the spectrum of target proteins, researchers are turning their attentions on the lysosome degradation system. Two marvelous approaches, termed LYTACs 21 and AbTACs 22, were reported to degrade secreted and cell-surface proteins by hijacking cell-surface lysosomal targeting proteins. Bertozzi's group established the first-generation LYTAC, M6Pn-LYTAC, combining the ligand of the cation-independent mannose-6-phosphate receptor (CI-M6PR) with an extracellular protein binder 21. Pioneering work from research groups of Bertozzi, Spiegel and Tang developed the second-generation LYTAC, GalNAc-LYTAC, with the advantage of cell-specific degradation by engaging the liver-specific asialoglycoprotein receptor (ASGPR) 23-25. Wells and colleagues used the AbTAC, a bispecific antibody, to expand the scope of targeted degradation via transmembrane E3 ligases 22. Up to now, the degradation role has been established in secreted protein apolipoprotein E4 (ApoE4) and membrane-bound proteins, including epidermal growth factor receptor (EGFR), CD71, programmed death-ligand 1 (PD-L1) 21, 22, etc. LYTACs are able to induce target protein degradation with a high degree of selectivity to POIs and tissues, while AbTACs exploit the new bispecific antibody's potential for protein degradation. Although substantial progress has been made in the development of TPD, the emerging degradation approaches demand high technical requirements and each faces its own set of challenges towards its way to the clinic. Hence, this review compares the emerging degradation platforms, summarizes the design, synthesis, and mechanism of LYTACs and AbTACs, and highlights their future directions and challenges.

## Overview of the novel degrader technologies

**Figure 1:**
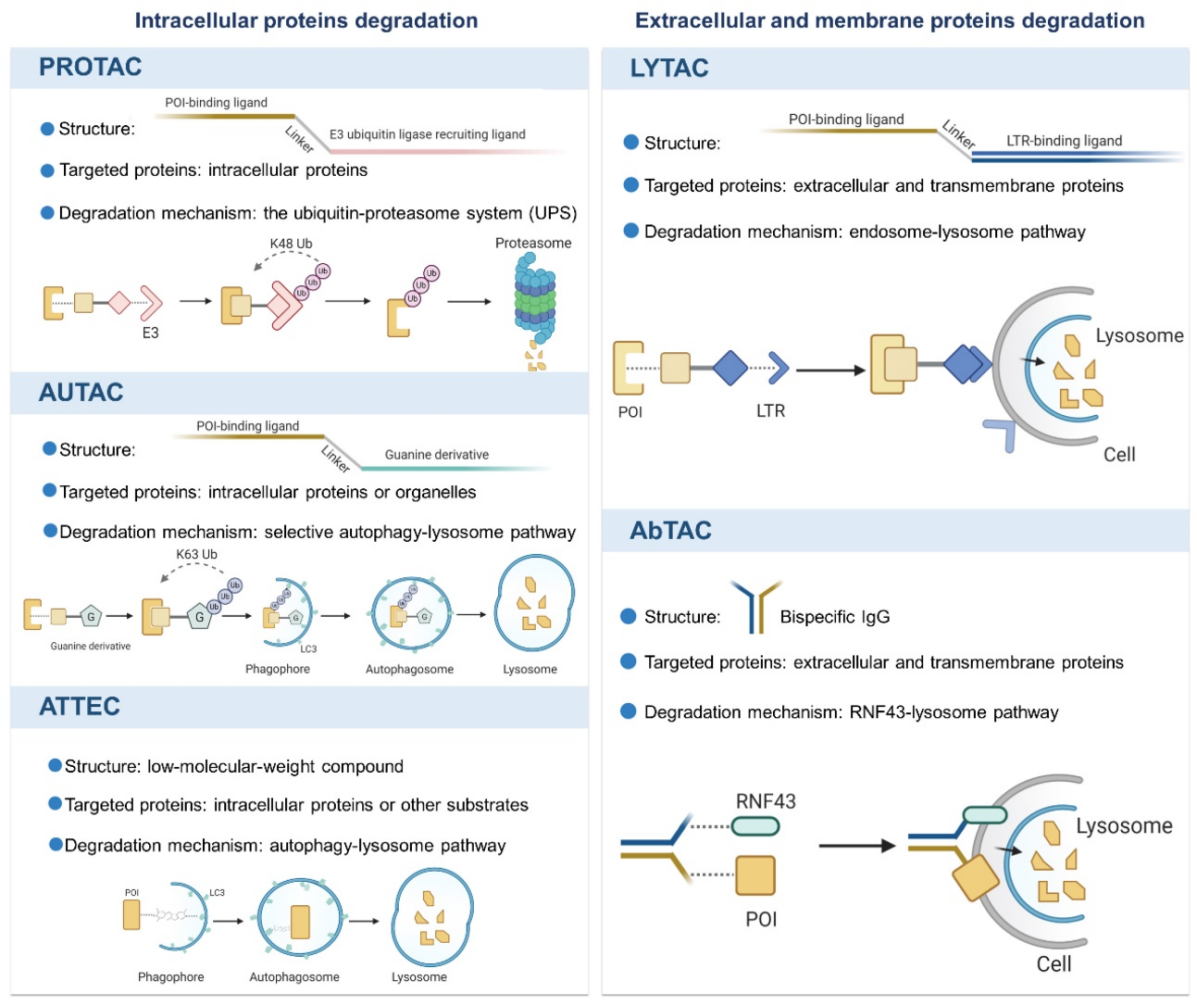
An overview of the novel protein degradation technologies. LYTAC and AbTAC utilizes lysosome system to degrade extracellular and membrane POIs. Intracellular POIs are targeted and degraded by PROTAC through the UPS. AUTAC and ATTEC technologies take advantages of autophagy-lysosome system to selectively degrade intracellular proteins and even organelles. Figure created with BioRender.com.

Over the last two decades, the breakthrough progress in TPD technologies not only provides useful tools for biological discovery, but also offers viable therapeutic candidates in clinic. We herein summarize the novel technologies with different mechanisms, as shown in Figure 1, and discuss their potential applications and limitations.

## Intracellular proteins degradation

### PROTAC

A PROTAC molecule consists of a targeting warhead for intracellular POI and an E3 ligase ligand for recruitment of E3, which are connected by a flexible linker 26. The formation of a POI-PROTAC-E3 ternary complex induces ubiquitination of the POI and subsequent its degradation cascade by the 26S proteasome 27. The PROTAC concept provides a powerful strategy for targeting those “undruggable” proteins that lack an active site for an inhibitor to bind or is not addressable by an inhibitor 28. Of note, event-driven degradation induced by PROTAC is catalytic and sub-stoichiometric due to its multiple cycles of degradation mechanism 29,30. Currently, a variety of PROTAC molecules have been designed and validated in preclinical settings and two oral PROTACs ARV-110 targeting AR (NCT03888612) and ARV-471 for ER (NCT04072952) have shown promising prospects in phase I clinical trials with good efficacy and safety profile 19,31,32. Despite the promising clinical data, PROTACs have certain limitations to target those “undruggable” proteins with large shallow surfaces as well as extracellular proteins 10,33-35. Small molecules and peptides are currently the main targeting warheads used by PROTAC design. However, those ligands have corresponding limitations. Among them, small molecules heavily rely on the binding pockets of POI 36, while peptide ligand application is limited to poor cellular membrane penetration and stability* in vivo* 37. Moreover, PROTAC has faced challenges such as further expansion of human E3 ligases, reducing off-target toxicity, and the optimal linker-length determination, which have hindered its development. For example, because only a handful of E3 ubiquitin ligases are available, the current application of PROTAC is restricted. Similar to PROTAC, other UPS-based modalities using limited E3, like molecular glues, dTAG, and Trim-Away, are under restrictions.

### AUTAC and ATTEC

In addition to the extensively utilized UPS in current TPD approaches, novel techniques such as AUTAC and ATTEC have been developed to modulate and control protein levels by harnessing the autophagy/lysosome pathway (reviewed in 38), which offer a glimpse into future possibilities 11,12,39.

An AUTAC molecule consists of a small molecular binder of target protein and a guanine derivative as a degradation tag to trigger K63 polyubiquitination (different from the K48 polyubiquitination triggered by PROTACs) 11. Ubiquitinated POIs are recognized by autophagy receptors such as p62/SQSTM1 and are linked to phagophores through the LC3-interacting region 40-42. Despite the unique advantages of AUTAC for its specific and broad degradation scope, the underlying mechanisms of selective autophagy and its effects on the overall cellular proteins remain unclear and require further investigation.

Similar to AUTAC based on autophagy-lysosome system, ATTEC is a linker compound that tethers the POI to the autophagosome by interacting with both POI and LC3 proteins 12. Owing to the advantages of its small size, ATTEC manipulates the protein levels more effectively. Meanwhile, it also reminds us that a largely unexplored area of compounds regulating therapeutically relevant proteins or other cytoplasmic substrates needs to be further exploited and clarified. These degraders all provide orthogonality and optimization for TPD platforms.

## Extracellular protein degradation

Despite the promising prospect of TPD strategy, non-cytosolic proteins lied beyond the scope of TPD for a long time, which limited their further application. Encouragingly, the novel technologies, LYTAC and AbTAC, have emerged to broaden the spectrum of protein targets.

### LYTAC

**Figure 2:**
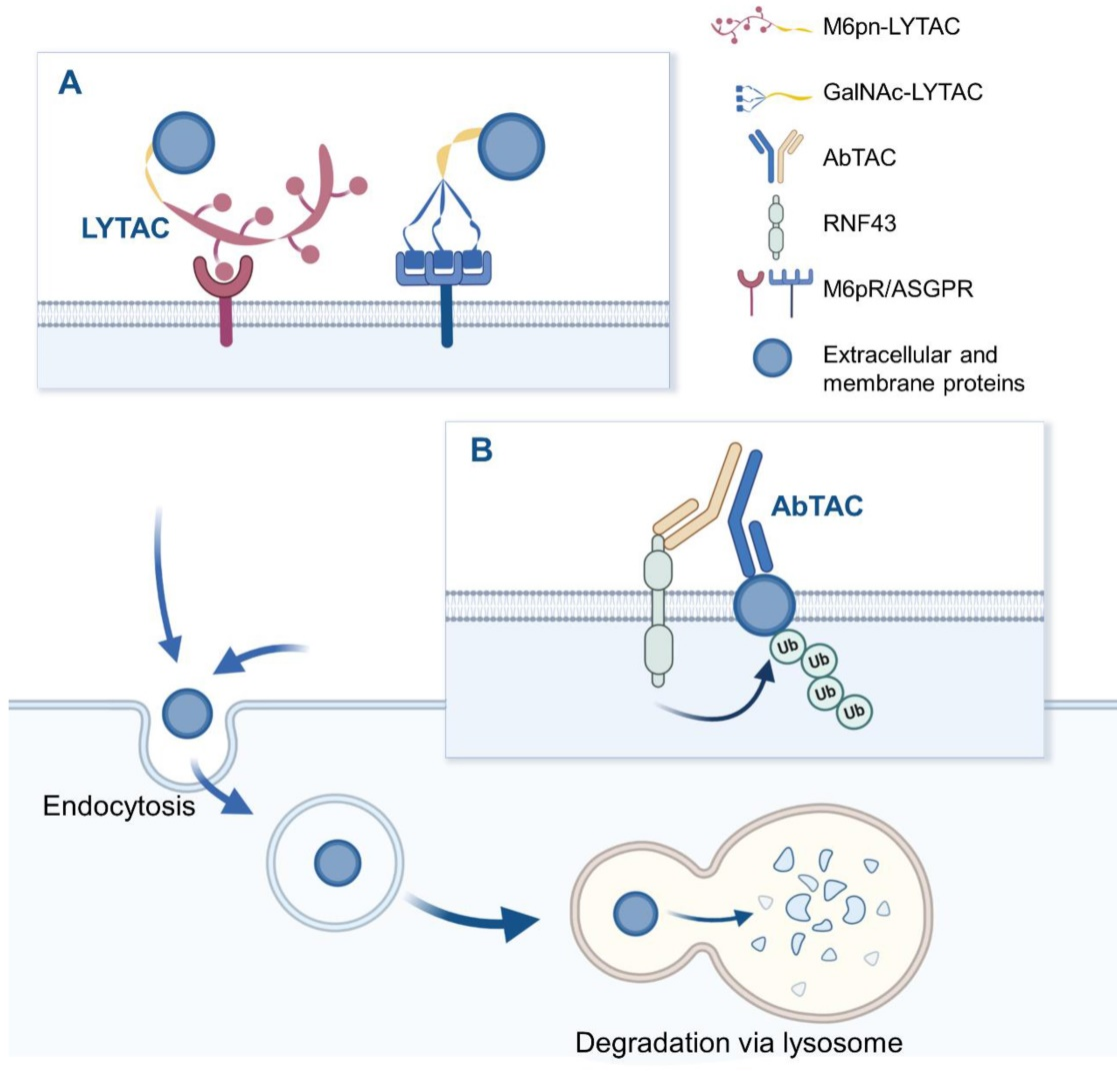
The schematic diagram of LYTAC and AbTAC. (A) M6Pn-LYTAC targets extracellular or membrane protein and is recognized by lysosome shuttling receptor CI-M6PR at the cell surface, to form ternary complex, while GalNAc-LYTAC binds target protein and liver cell-surface ASGPR simultaneously. The resulting complex is engulfed by the cell membrane, endocytosed into endosomes, and degraded in lysosomes. (B) AbTAC binds to RNF43 and cell-surface proteins simultaneously, inducing RNF43-AbTAC-protein complexes internalization and lysosomal degradation. Figure created with BioRender.com.

LYTAC is a novel technology that targets extracellular and/or membrane protein to induce degradation by harnessing the endosome/lysosome pathway. It is a bifunctional conjugate that simultaneously binds the extracellular domain of a target and a cell-surface lysosome-targeting receptor (LTR) to form a ternary complex, leading to protein internalization via clathrin-mediated endocytosis 43. After being engulfed, the complex successively passes through early endosome (EE) and late endosome (LE) where a low pH enables the complex to be dissociated 44. Subsequently, POI proceeds to lysosome to be degraded, while LTR is recycled into cell membrane via recycling endosome (RE). Degradation mechanism of LYTAC is shown in Figure 2. Notably, compared to POI inhibition, LYTAC directly exerts degradation effect on protein, and therefore avoids the potential activation of other downstream pathways that may be caused by inhibitors 21. Moreover, this degradation strategy prevents molecular compensation and cellular adaptation due to their higher depletion efficiency compared with genetic techniques like CRISPR-Cas9 45.

### AbTAC

Bispecific antibodies (bsAbs) refer to a large family of molecules that recognize two different epitopes or antigens 46. AbTAC is a fully recombinant bispecific immunoglobulins G (IgG) that can recruit transmembrane E3 ligases ring finger 43 (RNF43) 47 and cell-surface proteins simultaneously, inducing RNF43-AbTAC-protein complexes internalization and subsequent lysosomal degradation of POI 22, as shown in Figure 2. However, its mechanism of action is mainly remained elusive. Particularly, it remains unknown whether RNF43 ubiquitinates the intracellular regions of POI to induce endocytosis. Meanwhile, it needs to clarify what proteins are required for the AbTAC system and whether the RNF43-dependent degradation manner leads to other changes in cellular functions. Although there is no large cellular perturbation in whole-cell proteomics, the cell safety of AbTAC requires further proof 48. In addition, when screening AbTAC for optimal degradation efficiency, we should also take the RNF43 cell specificity and endocytosis kinetics into account.

To fully understand the discussed techniques and choose the appropriate one for the problem at hand, we compare the advantages and disadvantages of intracellular protein degradation strategies and the two extracellular protein degradation approaches (LYTAC and AbTAC), as shown in Table 1.

## Design and synthesis of LYTAC and AbTAC

To date, LYTAC and AbTAC have been the main two TPD technologies for extracellular and membrane protein degradation. Although they are recognized as tremendous milestones in the development of TPD, targeting extracellular protein degradation is still in its infancy. In this part, we mainly summarize the design principles, recent progress, and current synthesis strategies of LYTAC and AbTAC, providing theoretical basis for the future application. By focusing on the successive conceptual and technical innovations in this field, we hope to encourage and inspire further efforts to optimize current techniques and create new TPD methods.

### LYTAC

**Figure 3:**
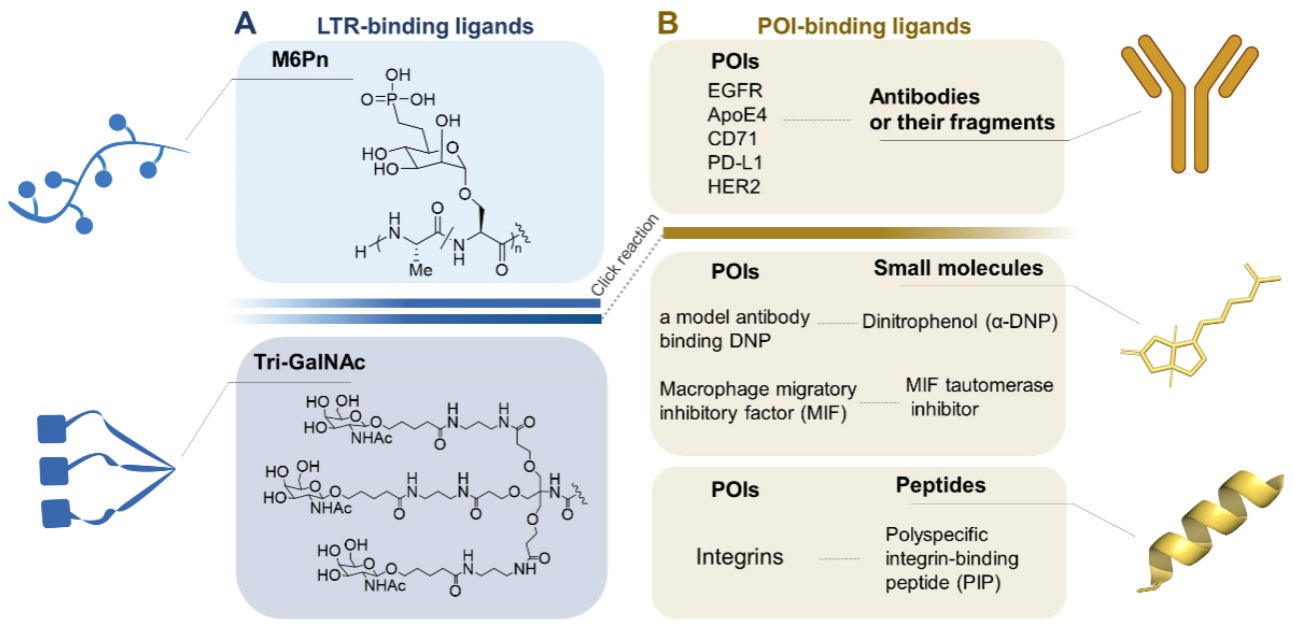
A toolbox of functional LYTAC. (A) The design of LTR-binding ligands. (B) The currently targeted extracellular and transmembrane POIs and their ligands. Figure created with BioRender.com.

LYTAC is designed with one end binding to the POI and the other end recognized by LTR. The incorporation of a ligand for efficient drug delivery via a LTR has been widely explored 54. Based on structure of two substrates and the certain degradation mechanism, LYTAC has been rationally designed and synthesized, as shown in Figure 3.

### LTR-binding ligands

#### PolyM6Pn

Cation-independent mannose-6-phosphate receptor (CI-M6PR), one member of cell-surface LTR families, can enclose hydrolases or other cargoes in endosomes, and subsequently deliver those proteins into lysosomes for depletion 55. Based on the structure of CI-M6PR, studies have demonstrated that CI-M6PR can bind with mannose-6-phosphate (M6P) glycoproteins 56. So far, M6P derivatives have been utilized in enzyme replacement therapies for lysosomal diseases or in neoplastic drug targeting usage 55. In order to increase affinity, stability, and avoid cytotoxicity, multiple M6P analogues were designed and tested, and an appropriate ligand M6Pn glycopolypeptide was obtained. Additionally, factors like length, chemical modification or side-chains should be further studied to achieve optimal CI-M6PR agonism 57.

M6Pn glycopolypeptides synthesis strategy starts with the conversion of mannose pentaacetate to M6Pn-NCA in 13 steps and ends with copolymerization of M6Pn-NCA to acquire poly(M6Pn) polypeptides 21. However, as a ubiquitous receptor, the expression levels of CI-M6PR in various cell types may impact LYTAC-mediated degradation efficiency 21, or result in potential off-target effects. Moreover, some studies indicated that CI-M6PRs are overexpressed in specific cancer types (such as breast cancer) 58, and play a role in regulating cancer cell growth and motility 59. However, whether this regulation effect is disrupted under the action of LYTAC is still unknown.

#### Tri-GalNAc

Unlike CI-M6PR, Asialoglycoprotein receptor (ASGPR) is a LTR which is highly expressed in hepatocytes 60. Due to the ligand specificity and the ability of supporting multiple rounds of uptake 61, ASGPR has enormous potentials for liver cell specific degradation of proteins. On the basis of structure studies, it is clear that glycoproteins bearing N-acetylgalactosamine (GalNAc) or galactose (Gal) ligands can be recognized by ASGPR and internalized via clathrin-mediated endocytosis 62. Indeed, GalNAc has already been applied as therapeutic nucleic acid agents in preclinical or clinical settings 63,64. Studies also revealed that triantenerrary GalNAc (tri-GalNAc) ligands engage ASGPR with higher affinity compared to GalNAc 65,66. Tri-GalNAc ligands are synthesized from peracetylated GalNAc to tri-GalNAc-DBCO in 8 steps, and then are conjugated to azides on non-specifically labeled antibodies 23. Tang's group directly used the commercially available Tri-GalNAc-COOH. After being converted to Tri-GalNAc-NHS (N-hydroxysuccinimide) ester, Tri-GalNAc ligands are conjugated with the lysine residues on the antibody 24. Compared to M6Pn, selectively delivering undesired proteins to liver by tri-GalNAc ligands is much safer than ubiquitously delivery of POIs to various types of cells 24. We compare the advantages and disadvantages of polyM6Pn and tri-GalNAc in Table 2.

### POI-binding ligands

The degradation ability of LYTAC also relies on the affinity between POI and its ligand. For targeting the significant disease-associative proteins, there are three choices for POI ligands, including antibodies, small molecules, and peptides 21,23-25. Although having specific affinity with POI, antibodies with high molecular weight have lower tissue permeability than small molecules in several cases 68. Take it further, the large molecular LYTAC labeled with an antibody needs to be miniaturized 52. With the development of structure biology and virtual screening, small molecular targeting warheads possessing high affinity are selected 69. Considering their inability to target “undruggable” proteins with large shallow surface, researchers have drawn their attentions towards specific binding peptides, which have advantages of lower production cost and amenability to chemical synthesis 70. Compared to small molecules, peptides possess greater potential in structural modification by point mutation or truncation 34,71,72. Generally, based on the crystal structure of endogenous complex of POI and binding protein, the key interacting residues can be analyzed to design the peptide targeting warheads 73. To fully understand those POI ligands, we compared the advantages and disadvantages between them as shown in Table 3.

### Construction strategy: Click reaction chemistry

Chemically, a LYTAC molecule can be assembled via azide-alkyne cycloaddition (click chemistry) between a TLR ligand and a POI ligand. For example, following modular design and synthesis, a POI ligand labeling with azide group is conjugated with a TLR ligand with alkynylation via copper-free strain-promoted click reaction, in turn, it also works that a POI ligand labeled with DBCO (dibenzocyclooctyne) or BCN can conjugate to an azide-labeled TLR ligand. After the conjugation reaction, the product is interrogated by native gel electrophoresis 21,23.

### AbTAC

#### A single-pass E3 ligase: RNF43

As previously described, the AbTAC acts by assembling separately expressed half IgGs to form a bispecific IgG for degrading cell-surface proteins via RNF43 22, which comprises a structured ectodomain, a transmembrane region, and an intracellular RING domain 46. It was documented that RNF43 is widely expressed in a variety of cancer cells, such as MDA-MB-231, HCC827, and T24, etc., which provides generalizability for the degradation of RNF43-driven AbTAC 22. In addition to that, RNF43 can negatively regulate the Wnt signaling pathway by ubiquitinating the Frizzled receptor, causing its endocytosis and degradation 76, which may open up possibilities to degrade the cell-surface POIs via endocytosis.

### Construction strategy: Knobs-into-holes (KIHs) technology

**Figure 4:**
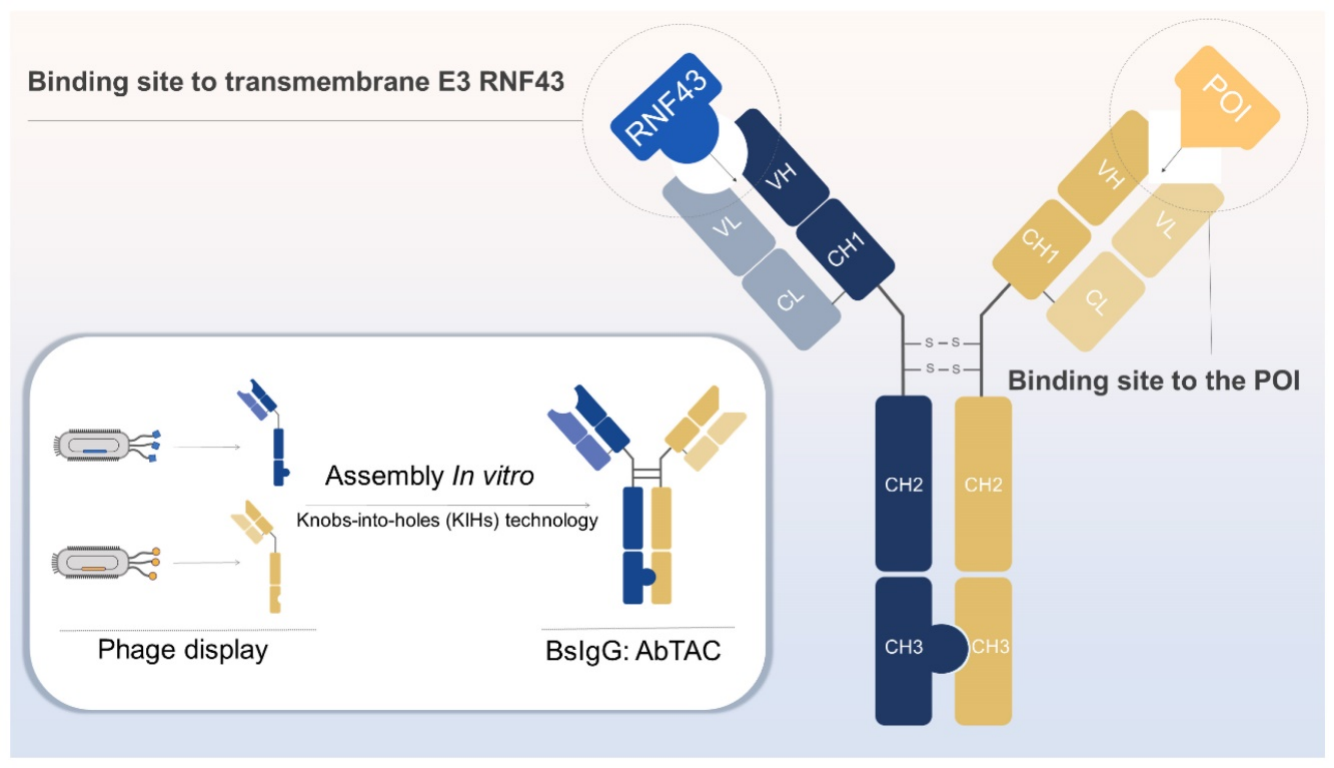
Generation of bispecific IgG AbTAC. The recombinant antibody for RNF43 and POI was generated using phage display. These two IgGs are assembled to form an AbTAC utilizing the knobs-into-holes technology. Figure created with BioRender.com.

The production of antibodies binding to RNF43 and POI begins with the phage display technique 77. After multi-round selection strategies, clones with high affinities were identified by sequencing and assessing the on-cell binding ability 22. Knobs-into-holes (KIHs) technology can prevent light chain mismatch pairing of half IgGs of anti-RNF43 and anti-POI, by creating either a “knob” or a “hole” in each heavy chain to promote heterodimerization 78. The composition of AbTAC and its generation strategy are shown in Figure 4.

## Future Perspectives

### Optimization of LYTAC and AbTAC

#### LYTAC

CI-M6PR is highly expressed in fetal and neonatal tissues but it decreases postnatally; however, it is overexpressed in some pathological conditions, such as fibrosis and some cancerous diseases 79-81. For example, CI-M6PR is induced in a number of human carcinoma cells, such as breast cancer, pancreatic cancer, gastric cancer, melanoma, and prostate cancer 80. Therefore, M6Pn-LYTAC is regarded as a more promising strategy for tumor therapy compared to GalNAc-LYTAC. Nevertheless, the construction of CI-M6PR-binding ligand, M6Pn glycopolypeptides, lacks precise manipulation, leading to the heterogeneity of products 21,57. It is adverse for the subsequent study on structure optimization and structure-activity relationship. For instance, LYTACs with various glycopolypeptide length was observed only minor differences in uptake efficiency, suggesting that there should be a shortest M6Pn length to achieve high degradation efficiency. More important, smaller molecular size means lower immunogenicity 21. Further, it was demonstrated that the projection topology of the saccharide units is of crucial importance for an efficient cell penetration mediated by CI-M6PR 59. The spatial arrangement of glycopolypeptide needs to be further investigated.

#### AbTAC

AbTAC represents a new archetype to LYTAC with the ability to deplete cell-surface proteins. Despite its great promise in protein degradation, it is unclear how AbTAC recruit membrane-bound E3 ligases to induce cell-surface POI degradation via lysosome pathway. Only when the detailed regulation process is elucidated, could we make good use of this degradation pathway better. Moreover, a potential problem is whether there is appropriate distance between half IgGs for binding of membrane-bound E3 ligase and POIs at the same time. In this case, we envision that a bifunctional peptide with two warhead and a flexible linker can be used to target transmembrane E3 ligase and POIs simultaneously. We can also utilize phage display to rapidly generate targeting peptide for membrane-bound E3 ligases with high affinity and high specificity 82. Meanwhile, there are so many existing tool peptides for disease related membrane proteins like PD-L1 83, CD47 84 and VEGFR 85. Intermediate linker between two peptides is chosen according to actual situation, resulting in a “peptide version” AbTAC. We hope that accessible molecule will inspire the advancement of extracellular proteins degradation as a novel class of drug candidates.

### Potential cell-surface lysosome targeting proteins

Currently, the number of E3 ubiquitin ligase ligands used in PROTAC technology is limited, which restricts their subsequent application and development. Also, it was verified that the loss of core components of E3 ligases leads to resistance to PROTACs 86. Therefore, rather than overusing the same lysosome shuttling receptors, we do need to exploit new lysosome targeting proteins. For example, CD22 recycling receptor specifically expressing on B-cells 87 or mannose receptors (MR, CD206) presenting on tumor-associated macrophages (TAMs) surface 88,89 may be may be good choices for cell-specific degradation. Similarly, ZNRF3, another cell surface E3 ligase, provides a potential opportunity for the development of AbTACs 47. In addition to cell surface receptors, intracellular lysosomal receptors, such as the lysosomal integral membrane protein (LIMP-2) and sortilin 90, might provide us with some enlightenments for targeting cytosolic proteins via lysosome system.

### New possibilities for clinic

#### LYTAC gives failed drugs another chance

LYTAC provides possibilities to change protein ligands into degraders. TPD strategy possesses kinetic advantages by binding to any “nook” or “cranny” on POIs with low dose 91,92. Generally, ligands that bind to the desired targets but were set aside because they could not adequately block protein function may be possible to be permitted as warheads of LYTAC. On the other hand, many preclinical or clinical 'occupancy-driven' molecules that have clear affinity with POI but not have gained FDA-approval due to their side effects can also be the warheads of LYTACs.

#### Multi-headed LYTAC and AbTAC-drug conjugates

Drug resistance and relapse are two major obstacles in cancer treatment 93, which can be avoided by rational polytherapy to target distinct pathogenetic mechanisms 94. Recently, a series of PROTACs (MT-802, SJF620, and L18I) have been reported that they can overcome the resistance to ibrutinib induced by BTK mutations 95. Based on this fact, we hypothesized that the “star molecule” multi-headed LYTACs might be able to overcome resistance with high efficiency and low toxicity 96,97.

Antibody-drug conjugates (ADC) are monoclonal antibodies conjugated to cytotoxic agents by environment-responsive linkers, which enable traditional drugs to have high tumor specificity and potency 98. Five ADCs have since achieved FDA approval and more than 40 are now in or nearing clinical trials 99,100. Here, we speculate the possibility of forming an AbTAC-drug conjugate (ATDC) as a multi-target agent. Ideally, after rational design of AbTAC linked to a drug, ATDC is able to degrade membrane protein to block the upstream signal, and further be internalized rapidly to deliver the linked drug intracellularly. Additionally, it requires that the linker is stable in blood circulation yet is readily cleavable at the target site. Future studies in MOA of AbTAC will possibly help to further develop AbTAC as a mature technology and discover effective multi-target agent.

#### Delivery nanosystem

Because LYTACs are macromolecules different from those of traditional protein-targeting small molecules, their aqueous solubility, drug delivery, and pharmacokinetics, and adverse reaction remain elusive. We estimate that the universal expression of cell-surface LTR and the instability of LYTACs may cause off-target effect and short drug half-life. Nanoparticles (NPs) have emerged as a powerful drug-delivery tool to enhance the specificity of drug actions, while reducing the systemic side effects 101. For instance, The ARV-loaded NPs successfully improved the solubility, permeability, pharmacokinetics, and delivery of ARV, showing promising anticancer activity. Moreover, surface modification of PLGA NPs with PEG can impart high serum stability and prolonged half-life to PROTAC 102,103. To ensure PROTAC delivery at the desired site in the required proportions, Ocaña and colleagues established PROTAC-loading NPs conjugated with antibody trastuzumab (selectively binds to the extracellular domain of HER2) to improve target selectivity, and cytotoxic efficiency for the treatment of breast cancer 104. According to these cases of PROTAC-loading NPs, we propose that LYTAC-loading NPs might enable prolonged circulation and targeted delivery through elaborate design. Besides, “passively” targeted NPs are the most extensively explored strategy targeting cancer, by utilizing the enhanced permeability and retention (EPR) effect 105. Moreover, stimuli-responsive NPs based on extrinsic stimuli have reached clinical trials and received approval 106. We believe that it is promising to explore the feasibility and application of LYTACs delivery nanosystem.

## Conclusion

Nowadays, TPD has been recognized as a game-changing strategy by inducing the degradation of the 'undruggable' targets and PROTACs have broken the classical Lipinski's rule of five 107. However, the substrates of PROTACs are limited to intracellular proteins through proteasomal clearance. LYTACs and AbTACs have emerged as potential therapeutics by taking advantage of the lysosome system to degrade disease-relevant extracellular and membrane proteins. Notably, there are also challenges in this nascent field, such as limited studies on length of linker, structure optimization, ternary complex equilibria, and pharmacokinetics. The critical next step is to develop them into mature technologies through interdisciplinary collaboration, which can provide more solutions. For example, synthetic procedure of M6Pn glycoproteins is interminable and inefficient. To increase efficiency, we can develop CI-M6PR-binding oligopeptides to replace the long M6P chain or simplify synthetic steps in chemistry, or search for other TLRs from a biological standpoint. Furthermore, the screening of LYTACs generally depends on measuring protein levels through western blot assay or mass spectroscopy, which is time-consuming. Development of computational tools that provide high-throughput methods to design and screen LYTACs may be a trend 108. Moreover, the concept of degradation strategy offers possibilities to degrade RNA genome, further establishing more therapeutic tools at the level of genetic degradation 109,110.

In conclusion, by focusing on TPD strategies towards extracellular and membrane proteins, we hope to provide readers with a resource to help navigate in this booming field, and inspire further efforts to create new degradation modalities.

## Figures and Tables

**Table 1:** Advantages and disadvantages of five TPD technologies

POIs	Techs	Advantages	Disadvantages
**Intracellular proteins**	PROTAC	Clear mechanisms of action 49; event-driven action 50; catalytic degradation activity 51;	Limitation to cytosolic domain of proteins 36; In a proteasome-dependent manner 45; Poor cellular penetration needs to be solved 36
	AUTAC	Targeting organelles and intracellular proteins; selective autophagy 11,39	Degradation mechanisms need further investigation 11,39
	ATTEC	Low molecular weight; selective degradation; Drug-like property 12	Hard to design 12
**Extracellular and membrane-bound proteins**	LYTAC	Targeting extracellular and membrane-bound proteins 21; high selectivity in POIs and cell types 23,24	Poor tissue permeability 21; Complex synthesis of LTR ligands M6Pn 21; Possible immune response *in vivo* 52; Lack of studies
	AbTAC	Targeting membrane proteins; high bispecificity 22	High cost; Potential immunogenicity 53; Unclear endocytosis mechanism 22

**Table 2:** Advantages and disadvantages of polyM6Pn and tri-GalNAc

	Advantages	Disadvantages
**PolyM6Pn**	High affinity 59; overexpression in specific cancer cells 58	Complex inhomogeneity of structure 68; complicated synthesis process 21
**Tri-GalNAc**	Homogeneous structure 24; cell type-specific degradation 23,24	Limitation on liver cells 23,24;

**Table 3:** Advantages and disadvantages of POI ligands

	Advantages	Disadvantages
**Antibodies**	Specific affinity with POIs 74	High cost; low stability and tissue permeability 21; potential immunogenicity 52
**Small molecules**	High stability 75; good penetrability 25	Inability to target “undruggable proteins” 25
**Peptides**	Low cost; easy to synthesise 61	Low stability; limited penetrability 36

## Abbreviations

TPD: targeted protein degradation
PROTAC: PROteolysis TArgeting Chimera
POI: protein of interest
LYTAC: LYsosome-TArgeting Chimaera
AbTAC: Antibody-based PROTAC
Erα: estrogen receptor alpha
AR: androgen receptor
AUTAC: autophagy-targeting chimera
ATTEC: autophagosome-tethering compounds
dTAG: degradation tag
CI-M6PR: cation-independent mannose-6-phosphate receptor
ASGPR: asialoglycoprotein receptor
ApoE4: apolipoprotein E4
EGFR: epidermal growth factor receptor
PD-L1: programmed death-ligand 1
UPS: ubiquitin-proteasome system
LTR: lysosome-targeting receptor
EE: early endosome
LE: late endosome
RE: recycling endosome
bsAbs: Bispecific antibodies
IgG: immunoglobulins G
RNF43: cell-surface transmembrane E3 ubiquitin ligase ring finger 43
ZNRF3: cell-surface transmembrane E3 ubiquitin ligase zinc and ring finger 3
VEGFR: vascular endothelial growth factor receptor
M6P: mannose-6-phosphate
GalNAc: glycoproteins bearing N-acetylgalactosamine
Gal: galactose
tri-GalNAc: triantenerrary glycoproteins bearing N-acetylgalactosamine
DBCO: dibenzocyclooctyne
BCN: cyclopropane cyclooctyne
KIHs: Knobs-into-holes
MR: mannose receptor
TAM: tumor-associated macrophage
LIMP: lysosomal integral membrane protein
ADC: Antibody-drug conjugate
ATDC: AbTAC-drug conjugate
MOA: mode of action
NPs: Nanoparticles
